# Foot-and-mouth disease dynamics in multi-species livestock systems at the interface of African protected areas

**DOI:** 10.1186/s13567-025-01487-y

**Published:** 2025-03-19

**Authors:** Oriane Ploquin, Vladimir Grosbois, Mthabisi Ndlovu, Simbarashe Ndozore, Martin Munzamba, Emildah Porovha, Khanyile Nkomo, Oriane Basso, Gaelle Corbel, Richard Shumba, Masocha D. Mhlanga, Ellen Mwandirigana, Benjamin Musekiwa, Elijah Takayindisa, Anais Loisier, Hervé Fritz, Florian Liégeois, Alexandre Caron, Franck Prugnolle, Eve Miguel

**Affiliations:** 1 UMR MIVEGEC, Research Institute for Development (IRD), University of Montpellier, French National Centre for Scientific Research (CNRS), Montpellier, France; 2 UMR ASTRE, French Agricultural Research Centre for International Development (CIRAD), National Research Institute for Agriculture, Food and the Environment (INRAE), Montpellier, France; 3 Department of Wildlife Ecology and Conservation, Chinhoyi University of Technology, Chinhoyi, Zimbabwe; 4 Hwange Long-Term Socio-Ecological Research Site, Zone Atelier, Dete, Zimbabwe; 5 Department of Veterinary Services, Dete, Zimbabwe; 6 Department of Veterinary Services, Malipati, Zimbabwe; 7 Faculty of Veterinary Science-Biotechnology Centre, University of Zimbabwe, Harare, Zimbabwe; 8 UMR RECOVER, National Research Institute for Agriculture, Food and the Environment (INRAE), Aix-En-Provence, France; 9 IRL REHABS, International Research Laboratory, French National Centre for Scientific Research (CNRS), University of Lyon 1, Nelson Mandela University, George, South Africa; 10 Faculty of Social and Behavioural Sciences, University of Zimbabwe, Harare, Zimbabwe

**Keywords:** Multi-host pathogen, wildlife-livestock interface, cattle, goat, Zimbabwe

## Abstract

Many pathogens have the capacity to infect multiple hosts. Multi-species epidemiological systems are characterized by populations that interact and perform different functions in pathogen transmission and maintenance. This study investigated the epidemiological dynamics of foot-and-mouth disease (FMD) virus in cattle and goats and their respective functions in disease circulation within sympatric livestock populations adjacent to wildlife areas in Zimbabwe. Through year-long longitudinal serological monitoring, the spatial distributions of FMD antibodies and associated risk factors were examined. The results revealed significantly greater FMDV seroprevalence in cattle than in goats, with serostatus in cattle being influenced by proximity to wildlife areas. In contrast, goats presented a lower seroprevalence, less variation among age groups, and no association with proximity to protected areas. On the other hand, clustering analysis indicated the absence of clustering of seropositive individuals at the herd scale, suggesting low levels of virus transmission between animals belonging to the same herd in both species. These findings highlight the significance of context-dependent interactions among hosts, particularly with wildlife. This study emphasizes the necessity of comprehensive surveillance and strain identification across multiple sympatric species, both wild and domestic, for the effective management of multi-host pathogens. In conclusion, this research contributes to understanding the complex dynamics of FMD transmission in rural areas in Zimbabwe and emphasizes the importance of tailored surveillance strategies in diverse ecological settings.

## Introduction

Many pathogens that affect animals can infect multiple hosts across wildlife, livestock, and human populations [1, 2]. These multi-host systems are characterized by complex networks of transmission between sympatric host populations, which makes it difficult to manage the resulting diseases. Effectively managing infectious diseases necessitates a comprehensive understanding of the epidemiological functions of each species and each population within these multi-host systems [3]. The presence of a pathogen in different host populations leads to a categorization of the role of species within epidemiological compartments. From reservoir populations, in which the virus persists, to target populations through intermediate hosts, the epidemiological functions of each individual are the result of the competence of the species for the pathogen [4] and its exposure to the risk of infection [5]. The dynamics of infectious diseases in a population depend on interactions between and within populations [6, 7]. In sub-Saharan savannas, the progressive encroachment of both human agricultural and extractive activities into natural habitats has expanded the areas of contact between wild and domestic animals [8]. These interfaces are influenced by the combined effects of animal husbandry practices and protected area management and lead to increased human/domestic animal/wildlife interactions that can translate into affected levels of interspecies transmission of pathogens [9–11].

Foot-and-mouth disease (FMD) is a highly contagious disease of cloven-hoofed animals that affects livestock worldwide [12]. The causative agent of FMD if the foot-and-mouth disease virus (FMDV) from the *Aphthovirus* genus in the *Picornaviridae* family. Seven serotypes (A, O, C, Asia1, and South African Territories 1, 2, and 3) circulate over a wide geographic range and differ in their virulence and pathogenicity, requiring context-specific management strategies [13]. The main wild reservoir host of FMDV is considered to be the cape buffalo (*Syncerus caffer caffer*) in southern Africa. A wide variety of other wild African ungulate species nevertheless have the potential for virus shedding [13–15]. Understanding the role of domestic and wild populations in the maintenance and transmission of pathogens is key for managing wildlife/livestock interfaces. In Southern Africa, one narrative states that the virus is primarily maintained by wildlife and spills over to cattle populations where it cannot be maintained owing to veterinary control of outbreaks when vaccination breaks the transmission chain [14, 16, 17]. Another narrative has emerged more recently on the basis of some forms of silent circulation of FMDV in cattle [17, 18]. Under this latter scenario, FMDV can be maintained in cattle populations, and the role of wildlife becomes either marginal or additional to the role of cattle [19]. Therefore, in multi-host systems at the wildlife/livestock interface, exploring the role played by different sympatric wild and domestic populations to decipher FMD epidemiology is crucial.

Following an infection, an animal can become a carrier [20], a status that entails the persistence of the virus in the host’s system without any noticeable symptoms. All hosts of FMDV have the potential to become carriers, but cattle stand out owing to their stronger potential for virus maintenance, which can even reach the reservoir stage in populations continually exposed to the same virus strains [1, 21]. Small ruminants, such as goats and sheep, also have the potential to act as both susceptible and competent hosts for FMD virus in southern Africa [22, 23]. While they primarily manifest subclinical infection cases [24–26] and appear to be less efficient hosts for transmission than cattle are [27], they remain noteworthy for their ability to shed the virus into the environment [28]. Experimentally, goats are susceptible to FMDV, can harbour the virus for up to 4 months post-infection, and have been suggested to have contributed to previous FMD outbreaks worldwide [28, 29].

By exhibiting mild or no symptoms, the circulation of the virus in these small ruminant populations can remain silent, undetected and uncontrolled [30]. In addition, goats are the predominant livestock population in southern Africa and could constitute a large epidemiological population for FMD [24]. Consequently, numerous studies emphasize the critical need to investigate the role of small ruminants in the circulation of FMDV [31–33].

In Zimbabwe, the boundary between protected and communal lands is mostly without the demarcation of a physical fence, making the interface relatively porous and not restricting the movement of animals and contacts between sympatric wild and domestic species [7, 34]. Importantly, however, veterinary fence construction projects were initiated, notably at the interface of Gonarezhou National Park in 2005. These fences are rapidly destroyed by elephant or human activities and the lack of local resources to maintain them [35, 36]. Previous studies in the area have confirmed the circulation of FMDVs among cattle populations in these border zones with wildlife. Specifically, proximity to protected areas and, more finely, contact with reservoir populations such as buffalo have already been identified as key risk factors in disease transmission [7, 16, 37].

In this study, we present the monitoring of FMDV antibodies in livestock populations (i.e., goats and cattle). This is the first longitudinal survey of goats and cattle simultaneously in 3 socio-ecosystems in Zimbabwe (i.e., Hwange, Gonarezhou and Kruger). We analysed the risk factors influencing the occurrence of exposure risk in animals. This approach facilitates a deeper understanding of these multi-host pathogen systems and the role of each host. This study was designed to characterize the relative roles of goats and cattle in the transmission of FMDV in livestock populations in contact with wild hosts.

## Materials and methods

### Study system

**Figure 1:**
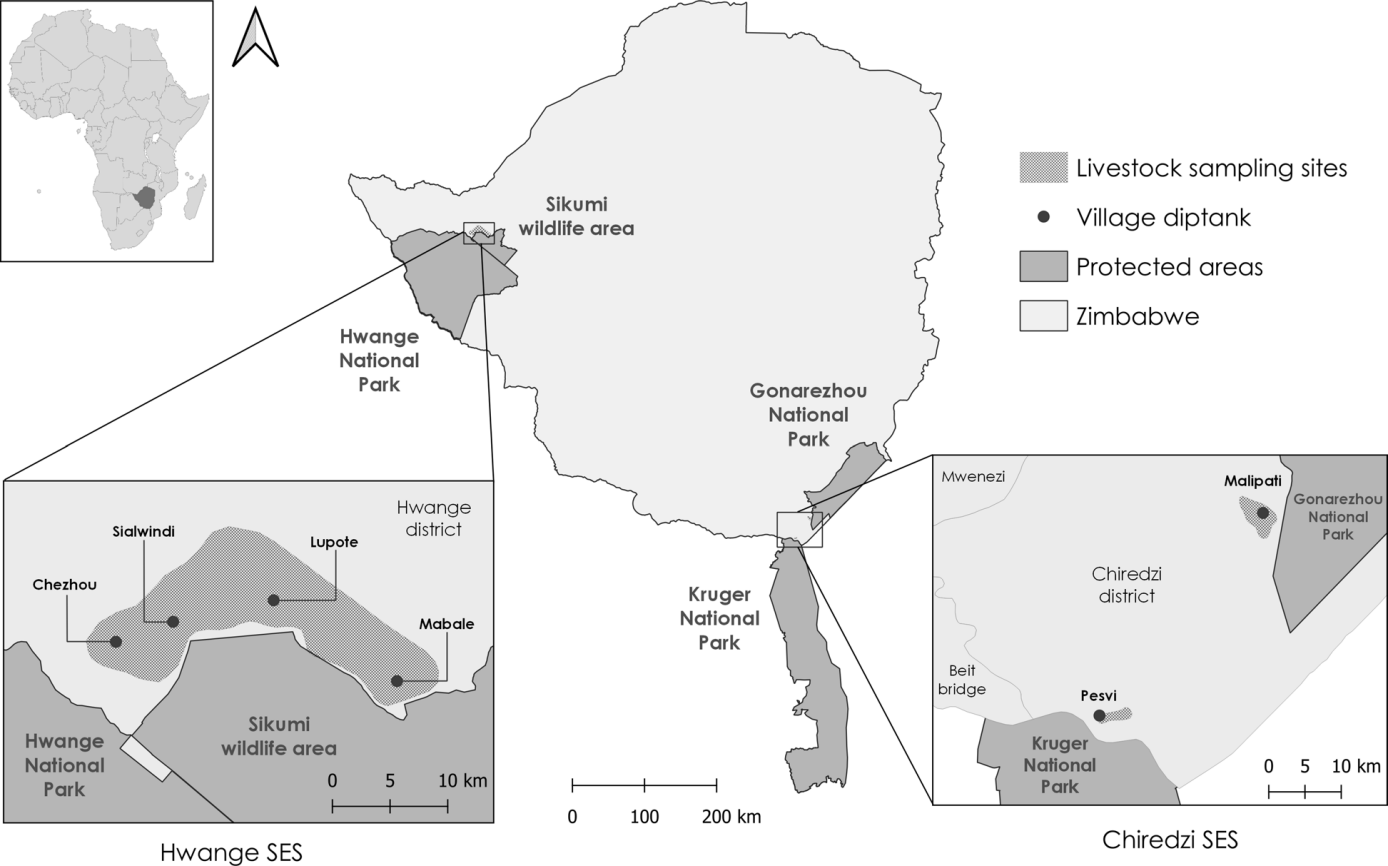
**Locations of the 3 livestock sampling sites and the nearest protected/wildlife areas in Zimbabwe grouped into 2 socio-ecosystems (SES): Hwange SES in the northwest of the country and Chiredzi SES in the southeast**. The sampling zones correspond to a buffer of the location of the farms where the sampled animals’ households reside (QGIS).

Our study encompasses two districts of Zimbabwe (i.e., Hwange and Chiredzi) previously studied for FMDV circulation [10, 16, 38] characterized by three porous interfaces between communal areas and national parks (NP) in TFCAs (TransFrontier Conservation Areas): Dete/Hwange NP in Kavango-Zambezi TFCA, Malipati/Gonarezhou NP and Pesvi/Kruger NP in Great Limpopo TFCA (Figure 1). The three sites are characterized by a semiarid savanna biome and an arid hot steppe climate [39]. These NPs exhibit notable large mammal diversity [40, 41]. In this paper, the three interfaces are combined into two socio-EcoSystems (SES) described in this section.

The Hwange SES is located in the Hwange district in the Matebeleland North Province. It is neighboured by Hwange National Park (14 651 km^2^) and Sikumi Forest (1100 km^2^), both of which are wildlife conservation and tourism areas. Moreover, the hunting area depicted in Figure 1 is connected to the Sikumi Forest and is also characterized by the presence of wildlife similar to those found in neighbouring protected areas. The Sikumi Forest and the hunting concession are merged as the Sikumi wildlife area in this paper and are referred to as protected areas (PAs). Our survey covers 4 neighbouring villages in this area: Sialwindi, Chezhou, Lupote and Mabale (Figure 1). There has been no known record of outbreaks or even symptoms of FMD in this area for the last 30 years, but other neighbouring villages in Matebeleland North have been affected by FMD cases recently (district records). In the Chiredzi SES in the Southeast Lowveld, Malipati village is situated at the frontier of Gonarezhou National Park (5053 km^2^) (see Figure 1). The village of Pesvi is located further south in the Chiredzi district, bordering South Africa and specifically north of Kruger National Park (19 485 km^2^) (Figure 1). Owing to frequent FMD outbreaks in the area [16], with the last one occurring in December 2023 [42], biannual vaccination campaigns are organized, targeting 100% of the cattle in the villages bordering the PAs, excluding goats. A trivalent vaccine is used to protect against all three SAT strains (Aftovax provided by the Botswana Vaccine Institute).

### Survey of livestock

A longitudinal seroprevalence survey was conducted from April 2022 to June 2023 at the two study sites. In the Hwange district, the same animals were sampled every 2 to 3 months during the survey from April 2022 to June 2023, resulting in a total of 7 samples. In the Chiredzi district, one session took place at the peak of the dry season in November 2022, and another session took place at the end of the rainy season in April 2023.

The sampling strategy was to include as many cattle and goat herds as possible, given logistic constraints, to document in our sample as many different exposure conditions as possible and at the same time to include a subsequent number of animals from each sampled herd so that we would be able to assess within herd vs. between herd variability in serological status. An additional consideration was the total number of samples that could be collected and properly processed and analysed given the financial resources available. As a result of this strategy, we managed to sample 36 and 28 herds of cattle and goats, respectively, in Hwange SES and 19 and 20 herds of cattle and goats, respectively, in Chiredzi SES. On average, seven animals were sampled per herd for cattle in Hwange SES, six for goats, five for cattle in Chiredzi SES, and three for goats in Chiredzi SES.

Each sampled animal was randomly selected within its herd and tagged. Both males and females were included in the sample, and the animals selected were at least 6 months old for cattle and 3 months old for goats, a period after which maternal immunity protecting the young from FMD virus infection is supposed to disappear [43, 44]. The collection of samples from cattle took place at the diptank, a gathering place for cattle, which is equipped with the necessary structures for the smooth operation of the sampling with the collaboration of local veterinary services. Diptanks are a favoured location for contact between cattle, and on a regular basis, veterinary services organize “dipping” sessions where the animals pass through a water-filled corridor treated with acaricide. For goats, sampling took place directly at the farmer’s location, with the continued support of local veterinary services. During capture, approximately 10 ml of blood were sampled from the animal’s jugular vein. Serum was extracted from each animal’s blood via serum-separating tubes. The samples were stored at room temperature (e.g., 20–25 °C) until serum collection following decantation. The sera were then stored at −20 °C prior to analysis.

### Detection of NSP antibodies

Antibodies specific to non-structural proteins (NSPs) of the FMD virus are detected through immunological assays (ELISAs). The NSP competition ELISA commercial test (ID Screen^®^ NSP competition, ID.vet, France), used for this study according to the manufacturer’s instructions, detects antibodies produced following natural exposure to FMDV infection, regardless of the virus strain. The manufacturer’s specification of high specificity implies that the accuracy of antibody detection is close to 100%. Therefore, we considered a positive test result as evidence of a past natural infection with the FMD virus in the animal. Importantly, the test does not detect maternally derived antibodies [45] or antibodies produced as a result of vaccination. The lifespan of antibodies varies depending on the virus strain that leads to the immune reaction and the host health status, but previous studies in the same cattle populations highlighted a loss of antibodies occurring 4–8 months following the seroconversion event [10].

### Factors considered likely to generate variation in serological status

The serological status at the animal level was characterized through the number of positive samples divided by the total number of samples tested for that animal (ranging from 1 to 7). Risk factors related to FMDV seroprevalence, such as the age and sex of the animal, were identified by local veterinary services. With respect to age, the animals were categorized as follows: juveniles (< 1 year), subadults (between 1 and 3 years), and adults (over 3 years). The Euclidean distance between each animal's homestead and the nearest boundary of the protected system was estimated via QGIS, which relies on GPS positions recorded in the field. The farms were categorized on the basis of their distance to the nearest protected areas. This variable was used as a proxy of the wildlife/livestock interface and potential contact with wild hosts for FMDV. In this area, farmers living close to the protected area boundary send their cattle to use available grazing within the protected area [7, 46]. Through engagement with local farmers and veterinary services, it was collectively decided that beyond a threshold of 2 km from protected areas, farmers were not frequently using the option of grazing inside the protected areas because of the distance to walk to the boundary. Thus, this 2 km threshold was used to classify farmers as using or not using protected areas. In Hwange SES, 202 animals live close to PAs, and 119 animals live farther than 2 km; in Chiredzi SES, 50 and 110 animals live close to PAs, respectively.

### Statistical model for variation in serological status

All the statistical analyses were conducted via R (version 3.3.0 +) and RStudio 2023 software. Only 105 transitions out of 1314 intervals and 33 transitions out of 608 intervals for serological status were observed in cattle and goats, respectively. It was thus considered that serological incidence and reversion events were not frequent enough to inform infection/exposure patterns. Instead, for each animal included in the survey, the records of their serology results across sampling sessions were pooled to compute a binomial outcome consisting of the number of positive antibody detection results over the total number of serological tests produced for that animal. This outcome variable was considered suitable for addressing variation in infection/exposure according to spatial and individual factors. A generalized linear mixed model (GLMM) including the main effects for all the explanatory variables and biologically meaningful level 2 and 3 interactions was fitted to the pooled antibody detection outcome variable described above using the *glmer* function from the *lme4* package (Table 1). The selection of the final model and significance of the variables were determined using the AIC and likelihood ratio test p value, respectively. As a first step, this modelling procedure was applied to the dataset, which included goat and cattle data from the two SESs, primarily to assess the interactions involving the species and SES variables. In a second step, separate models were fitted for each of the four species and SES-specific data subsets to assess the statistical significance of the explanatory variables independently for each species and SES as well as to produce for each species and SES variation patterns for the explanatory variables that are more accurate than those produced by the selected global model, which, for most of the explanatory variables, does not produce SES and species-specific parameters. All fitted models included a herd-by-species random variable (producing one random term per owner and species) to account for potential clustering of similar values of the outcome variable within herds of each species. The variance and intraclass correlation coefficient (ICC) of the pooled antibody detection outcome variable at the various clustering scales were estimated from random effects (herd and individual) models for each species separately using the package rptR.

**Table 1:** **Variables originally included in the model**

Level 1	Level 2	Level 3
Species	Species: sex	Distance to PAs: study site: Species
Sex	Species: age class	Species: sex: age class
Age class	Species: study site	
Study site	Species: distance to PAs	
Distance to the PAs	Age class: sex	
	Age class: study site	
	Age class: distance to PAs	
	Study site: distance to PAs	

### Description of within- and between-herds serological prevalence

To describe and compare the serological prevalence in cattle and goats within herds and between herds, a single binary serological status was derived from the set of serological results of each surveyed animal: an animal was considered positive if no negative serological result had been obtained for that animal or if at least two serological positive results had been obtained for that animal. This single binary individual serological status was then used to characterize each surveyed herd. As a first step, because the number of goats surveyed per surveyed herd was 4 whereas the number of cattle surveyed per surveyed herd was 6, 4 cattle per herd were randomly selected from the database so that herd status would be based on the same number of animals for cattle herds and goat herds. A herd was considered positive whenever at least one of the 4 surveyed animals in that herd was positive according to the single binary individual serological status described above. These herd status and individual status proxies were used to evaluate between-herd prevalence (proportion of positive herds) and within-herd prevalence (proportion of positive individuals in positive herds) for goats and cattle in Hwange and Gonarezhou.

## Results

### Longitudinal survey data

**Figure 2:**
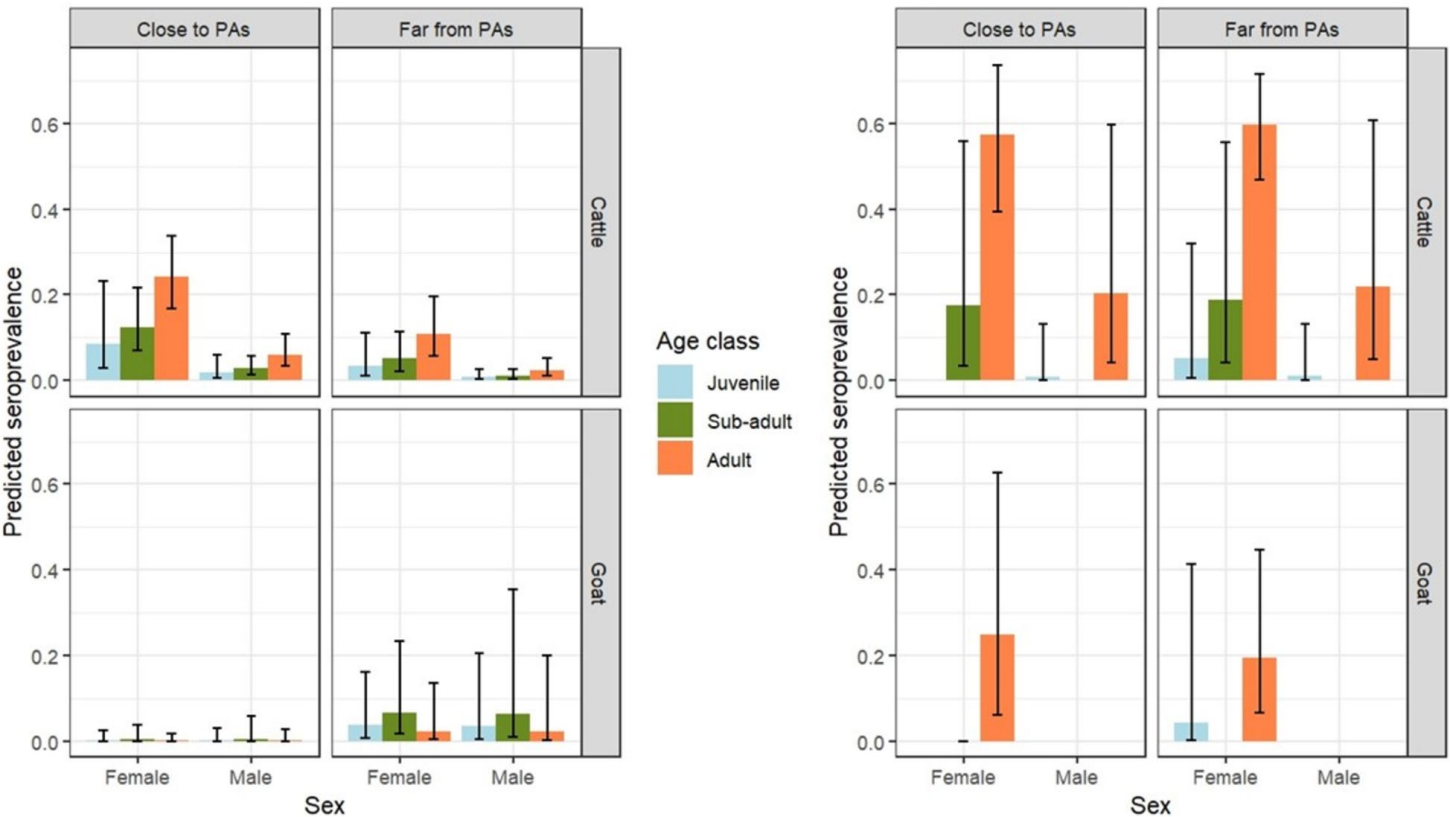
**Predicted seroprevalence at both study sites in terms of individual factors and the distance to PAs in (A) Hwange SES and (B) Chiredzi SES.** The bars indicate the seroprevalence estimates based on the fixed effects of the 4 sub-models run for each species at each study site. The error bars represent the standard error for a 95% CI.

Over the course of the 7 sampling sessions, 1792 samples were collected from 481 animals (313 cattle and 168 goats) (Table 2) belonging to 65 farmers (38 in Hwange SES, 27 in Chiredzi SES). Among these farmers, 15 exclusively owned cattle, 5 exclusively owned goats, and 41 owned both cattle and goats (28 in Hwange NP, 15 in Chiredzi SES). During each sampling session, 149 cattle and 64 goats were sampled on average in the Hwange SES, and 88 cattle and 47 goats were sampled in the Chiredzi SES. Among the 313 sampled cattle, 101 tested seropositive at least once during the study (34.5%). Among the 168 goats, 29 tested positive at least once (17.3%). Among the 55 sampled farms, 47 housed at least one cattle that tested positive at least once (85.5%), and 20 out of the 47 farms housing goats had at least once a seropositive goat tested (42.5%) (Figure 2).

**Table 2:** **Number of animals sampled at both study sites for all of the variables included in the model**

Study site	Hwange SES			Chiredzi SES			
	Close to PAs	Far from PAs		Close to PAs		Far from PAs	
*Distance to PAs*							
Cattle	135	75		33		70	
Female	82	41		30		65	
Juvenile	5	4		0		11	
Subadult	20	7		3		4	
Adult	57	30		27		50	
Male	53	34		3		5	
Juvenile	4	7		1		1	
Subadult	16	11		0		0	
Adult	33	16		2		4	
Goat	67	44		17		40	
Female	60	39		17		40	
Juvenile	15	11		0		12	
Subadult	33	19		2		0	
Adult	12	9		15		28	
Male	7	5		0		0	
Juvenile	5	3		0		0	
Subadult	1	1		0		0	
Adult	1	1		0		0

### Patterns of variation in FMDV seroprevalence

Generally, the serological prevalence was higher in the Chiredzi SES than in the Hwange SES, higher in cattle than in goats, higher in females than in males, and increased over successive age classes. There were, however, some variations in this general pattern, particularly for the effect of age on serological prevalence. Indeed, significant interactions between age and SES and between age and species were detected (*p* = 0.009 and *p* = 0.025, respectively; Table 3), indicating differences between species and between SES in the pattern of variation in serological prevalence according to age classes. Moreover, a significant interaction between species and distance to the protected area was detected, implying that serological prevalence varied depending on the distance to protected areas but differed across cattle and goats. The patterns of variation in serological prevalence revealed by the statistical models are reported in detail below.

**Table 3:** **Results of the generalized mixed model selected through AIC comparisons fitted to cattle and goats in both the Hwange SES dataset and the Chiredzi SES dataset**

Risks factors	Modalities	Estimate	Standard error	Delta AIC	*p* value (Chi^2^)
Full model				975.76	
*Intercept*		−1.3244	0.2535		
Species		–	–	–	–
	Cattle	*Ref.*			
	Goat	−2.8452	0.5718		
Sex				1020.44	8.4e−12***
	Female	*Ref.*			
	Male	−1.5256	0.2451		
Age class		–	–	–	–
	Juvenile	−1.2037	0.5363		
	Subadult	−0.7571	0.2922		
	Adult	*Ref.*			
Study site		–	–	–	–
	Hwange SES	*Ref.*			
	Chiredzi SES	2.1844	0.3666		
Distance to PAs		–	–	–	–
	Close (< 2 km)	*Ref.*			
	Far (> 2 km)	−0.7004	0.3777		
Species: age class				979.10	0.025*
	Goat: Juvenile	1.5255	0.7386		
	Goat: Subadult	1.3200	0.5581		
	Goat: Adult	*Ref.*			
Study site: age class				981.20	0.009**
	Chiredzi SES: Juvenile	−2.1676	0.9195		
	Chiredzi SES: Subadult	−1.6115	0.9508		
	Chiredzi SES: Adult	*Ref.*			
Species: distance to PAs				981.45	0.005**
	Goat: close to PAs	*Ref.*			
	Goat: far from PAs	1.7504	0.6455		

Significance codes: 0 ***, 0.001 **, 0.01 *.

### Variation in serological prevalence according to individual characteristics

In general, serological prevalence estimates were at least twice as high for cattle than for goats (Figure 2). The only exception was for cattle and goats living far from the protected areas in Hwange, where serological prevalence estimates for cattle and goats were fairly similar (Figure 2). This difference in the pattern of variation in serological prevalence according to the species was substantiated by a significant interaction between distance to the protected areas and species (*p* = 0.005 for the interaction between distance to protected areas and species; Table 3).

The serological prevalence increased more steeply with increasing age in Chiredzi SESs than in Hwange SESs and in cattle than in goats. The widest variation over age classes was thus observed in cattle from Chiredzi SES (*p* = 0.00001, Table 6), with a 2- to threefold increase over successive age classes (Figure 2). The serological prevalence increased more smoothly over successive age classes in cattle from Hwange SES (*p* = 0.0023, Table 4), with a 1.5- to twofold increase over successive age classes (Figure 2). Finally, the effect of age was only close to statistically significant in goats from Chiredzi (*p* = 0.058, Table 7) and was not significant in goats from Hwange (*p* = 0.20, Table 5). Whereas this last result was considered robust enough to indicate that serological prevalence did not vary noticeably across age classes in goats in the Hwange SES (Figure 2), for the Chiredzi SES, the number of goat samples analysed was low, with some poorly represented age classes (subadults), so that the analysis could not thoroughly address age variation in serological prevalence for goats in the Chiredzi SES (Figure 2).

**Table 4:** **Results of the final generalized mixed model fitted to the cattle in the Hwange SES data subset**

Risks factors	Modalities	Estimate	Standard error	Delta AIC	*p* value (Chi^2^)
Full model				559.98	
*Intercept*		−1.1419	0.2405		
Sex				602.16	3.002e−11 ***
	Female				
	Male	−1.5957	0.2664		
Age class				568.14	0.002295**
	Juvenile	−1.2353	0.5661		
	Subadult	−0.8072	0.2949		
	Adult				
Distance to PAs				563.45	0.019339*
	Close (< 2 km)				
	Far (> 2 km)	−0.9776	0.4287		

Significance codes: 0 ***, 0.001 **, 0.01 *.

**Table 5:** **Results of the final generalized mixed model for goats in the Hwange SES data subset**

Risks factors	Modalities	Estimate	Standard error	Delta AIC	*p* value (Chi^2^)
Full model				130.42	
*Intercept*		−6.28341	1.29680		
Sex				128.42	0.97147
	Female				
	Male	−0.02739	0.76690		
Age class				129.67	0.19749
	Juvenile	0.45799	0.79331		
	Subadult	1.07913	0.71474		
	Adult				
Distance to PAs				134.21	0.01616 *
	Close (< 2 km)				
	Far (> 2 km)	2.56324	1.21012		

Significance codes: 0.01 *

For cattle, serological prevalence estimations varied significantly according to the sex of the animal, and estimations for female individuals were approximately twice as high as those for male individuals (*p* < 0.001 for Hwange SES, Table 4; *p* = 0.04 for Chiredzi SES, Table 6, Figure 2). Although the interaction between sex and species was not selected in the model fitted to the full dataset, it was clear from the models fitted to the species and SES data subsets that serological prevalence did not vary to a great extent as a function of sex in goats (*p* = 0.83 for Hwange, Table 5; *p* = 0.77 for Chiredzi, Table 7, Figure 2).

**Table 6:** **Results of the final generalized mixed model fitted to the cattle in the Chiredzii SES data subset**

Risks factors	Modalities	Estimate	Standard error	Delta AIC	*p* value (Chi^2^)
Full model				201.21	
*Intercept*		0.2950	0.3734		
Sex				203.51	0.03809*
	Female				
	Male	−1.6624	0.8826		
Age class				219.96	1.151e−05***
	Juvenile	−3.2792	1.0674		
	Subadult	−1.8498	0.8698		
	Adult				
Distance to PAs				199.26	0.82652
	Close (< 2 km)				
	Far (> 2 km)	0.1000	0.4546		

Significance codes: 0 ***

**Table 7:** **Results of the final generalized mixed model fitted to the goats in the Chiredzi SES data subset**

Risks factors	Modalities	Estimate	Standard error	Delta AIC	*p* value (Chi^2^)
Full model				90.786	
*Intercept*		−1.1093	0.8662		
Age class				92.486	0.05784
	Juvenile	−1.6739	1.3867		
	Subadult	−18.8383	840.3272		
	Adult				
Distance to PAs				88.872	0.76991
	Close (< 2 km)				
	Far (> 2 km)	−0.3151	1.0911		

### Variation in serological prevalence according to SES and spatial characteristics

As substantiated by the highly significant interaction between age and SES (*p* = 0.009, Table 3), the magnitude of the difference in serological prevalence between the Chiredzi and Hwange SESs varied, particularly as a function of the age class of the animals considered. However, for juveniles, the estimated serological prevalence for Chiredzi SESs was less than twice as high as the estimated serological prevalence for Hwange SESs, which was up to ten times as high for adults (Figure 2).

Distance to the protected area influenced serological prevalence but differently depending on the species (*p* = 0.005 for the interaction between distance to protected areas and species; Table 3). For cattle, particularly in the Hwange SES (*p* = 0.02 for the effect of distance to protected areas for cattle in the Hwange SES, Table 4), serological prevalence estimations were twice as high for cattle living less than 2 km away from a protected area than for cattle living more than 2 km away from a protected area (Figure 2). The opposite pattern was observed for goats in Hwange SES (*p* = 0.02 for the effect of distance to protected areas for goats in Hwange SES, Table 5), where serological prevalence estimations for goats living less than 2 km away from a protected area were close to zero (less than 0.01) compared with estimations of approximately 0.05 for goats living more than 2 km away from a protected area. Although the interaction between distance to protected areas and SES was not selected in the model fitted to the full dataset, it was clear from the models fitted to the species and SES data subsets that the serological prevalence in the Chiredzi SES did not vary to a great extent as a function of distance to the protected areas (*p* = 0.83 and *p* = 0.77 for cattle and goats, respectively; Tables 6 and 7, Figure 2).

### Patterns of clustering in seroprevalence

For the two species and study sites, the individual intraclass correlation coefficients were high and significantly greater than 0 (Table 8). This is a clear indication that positive test results are clustered within individuals. Such aggregation is expected as long as it is assumed that antibodies produced following an infection persist for several months or years. In contrast, the within herd intraclass correlation coefficients did not significantly differ from 0, suggesting that positive individuals were not clustered within herds. By examining the proportion of herds with more than one positive result (indicating exposure to the FMD virus) and the serological prevalence within these exposed herds, it is clear that the prevalence between herds is much lower in goats than in cattle. However, the within-herd prevalence in positive herds is very similar between cattle and goats (Tables 9 and 10).

**Table 8:** **Animal-, farm- and SES-level sero-investigation of animals that were positive at least once during the survey**

Species	Hwange SES		Chiredzi SES	
	Cattle	Goats	Cattle	Goats
Intra-individuals	0.661 [0.627, 0.745]	0.644 [0.653, 0.801]	0.495 [0.263, 0.67]	0.731 [0.83, 0.998]
Intra-herds	0 [0, 0.007]	0.011 [0, 0.032]	0.012 [0, 0.153]	0 [0, 0.044]

**Table 9:** **Intra- and inter-herd prevalence in both species**

Species	Hwange SES			Chiredzi SES		
	Frequency of individuals with > 1 positive/only 1 positive/no positive serological result	Frequency of herds with > 1 positive/only 1 positive/no positive serological result	Mean frequency (overall frequency) of positive results in herds with > 1 positive serological result	Frequency of individuals with > 1 positive/only 1 positive/no positive serological result	Frequency of herds with > 1 positive/only 1 positive/no positive serological result	Mean frequency (overall frequency) of positive results in herds with > 1 positive serological result
Cattle	33/21/156 (16%/10%/74%)	22/8/6 (61%/22%/17%)	0.21 (0.21)	33/25/51 (30%/23%/47%)	16/2/1 (85%/10%/5%)	0.53 (0.55)
Goat	7/8/96 (6%/7%/87%)	5/5/18 (18%/18%/64%)	0.25 (0.23)	7/8/48 (11%/13%/76%)	7/4/9 (35%/20%/45%)	0.60 (0.54)

**Table 10:** **Intraclass correlation coefficients (ICCs) for serological results at the individual and herd levels**

Species	Hwange SES		Chiredzi SES	
	Cattle	Goats	Cattle	Goats
Intra-individuals	0.661 [0.627, 0.745]	0.644 [0.653, 0.801]	0.495 [0.263, 0.67]	0.731 [0.83, 0.998]
Intra-herds	0 [0, 0.007]	0.011 [0, 0.032]	0.012 [0, 0.153]	0 [0, 0.044]

### Association of seroprevalence in cattle and goats

In a mixed model including a farm-by-species random effect, the correlation coefficient between the seroprevalence farm random terms of cattle and goats sharing the same farm was −0.15, and this correlation was not statistically significant (*p* value = 0.339).

## Discussion

Control of FMD is hampered by the high contagiousness of the disease and the multitude of animal species capable of participating in its spread, especially in Africa. In Zimbabwe, the FMD control strategy consists of restrictions on animal movements and routine vaccination in high-risk areas identified on the basis of outbreak records and risk factor assessments. According to this strategy, rural areas at the border of protected areas are considered red zones [42], even when FMD symptoms are not reported because viral circulation can occur asymptomatically [17]. Because the epidemiology of FMD remains poorly documented in local contexts and human resources to implement control measures are scarce, FMD control is not fully successful, and the inability to prevent FMD outbreaks in livestock has negative impacts at the local and national levels [47, 48].

The present study investigated FMDV serological prevalence variation patterns in livestock species at two study sites close to protected areas harbouring wildlife species in Zimbabwe. The originality of our approach lies not only in the simultaneous epidemiological monitoring of goats and cattle sharing the same environment but also in the inclusion of two study areas characterized by contrasted livestock/wildlife interfaces and differing FMD epidemiological dynamics, as reflected by the frequent reporting of symptomatic cases in cattle in only one of the two study areas. The approach consisted of describing the patterns of variation in serological prevalence within and between study areas and species and according to individual characteristics and then suggesting plausible interpretations of these patterns with regard to mechanisms of transmission between wild and domestic animals, as well as within cattle and goat populations. The ultimate objectives were to gain insight into the role of cattle and goats in wild/domestic multi-host systems where contact between wild and domestic species can occur.

A serological survey was used to document the incidence of FMDV. Indeed, in contexts where viral strains do not consistently cause clinical symptoms, epidemiological investigations through serology can serve as an alternative to detect virus circulation. While the direct detection of the virus indicates an ongoing infection, the detection of antibodies indicates an infection at an undetermined time in the past, and serological prevalence levels are assumed to reflect the frequency and extent of circulation over time. The serological approach targeting antibodies used in the present study is thus considered reliable and is also less expensive than molecular approaches targeting viruses. Our results suggest that (i) cattle and goats carry detectable antibodies against FMDV without showing signs of infection during our longitudinal survey; (ii) the serological prevalence was higher in cattle than in goats, higher in females than in males, and increased over successive age classes; (iii) both species had higher seroprevalence rates in the Chiredzi SES than in the Hwange SES; (iv) the serological prevalence in cattle is higher close to the protected areas (PAs) than it is further from the PAs in Hwange SES but not in Chiredzi SES; (v) the serological prevalence clustering pattern differed between goats and cattle; and (vi) the serological prevalence in goats and cattle from the same farm was not correlated.

### Transmission from wildlife to domestic animals

A common assumption about the circulation of FMDV in wild/domestic systems is that the virus is transmitted from maintenance wild hosts (e.g., buffalo) to the most documented receptive species: cattle. In our study system, although the conservation areas hosting wildlife are protected zones with limited human activities, the authorities allow the local communities to use resources at the edge of PAs separated from the communal land by an open and porous border. Thus, communities use protected areas for collecting dry wood, and herders guide their cattle into PAs for grazing and water use [49]. The incursion into protected areas (PAs) and the subsequent proximity with wildlife can trigger FMDV spillover events at the wildlife/livestock interface, with more or less symptoms in the livestock population [50]. One objective of the present study was to assess this hypothesis via distance from farms to PAs as a rough proxy of the incursion of livestock herds in PAs. Indeed, previous research on livestock management practices in the Hwange area has shown that distance to PAs is a key factor in explaining farmers’ behaviour in relation to incursions into PAs [46]. In the present study, we indeed detected a higher FMDV serological prevalence in cattle living closer to PAs than in cattle living farther from PAs. This pattern was, however, observed only in the Hwange SES and was not observed in goats.

Compared with Hwange SES interfaces, Chiredzi interfaces exhibit notable differences, primarily due to the configuration of interactions between livestock and wildlife. Unlike the communal areas monitored at Hwange, where contact between cattle and buffalo largely occurs within buffer zones surrounding protected areas (PAs), in the Chiredzi SES, such interactions predominantly take place within communal areas themselves. Consequently, the interface between livestock and wildlife is even more permeable in Chiredzi SESs than in Hwange SESs in terms of the wildlife frontier [7]. Therefore, the distance to PAs may not adequately represent the extent of wildlife contact in Chiredzi SES, potentially explaining the absence of a relationship between FMDV serological prevalence and distance to PAs. Goats have more freedom of movement than cattle do, as they are not herded but rather move over shorter distances. Their enclosure is opened late in the morning, and they return only in the evening after freely roaming for water and food. Thus, although nothing prevents them from venturing into protected areas, they seem to avoid excursions out of villages, possibly due to the risk of predation by wild carnivores (e.g., hyenas, lions, and wild dogs) [51]. Given this behaviour, a weaker relationship between FMDV serological prevalence and distance to PAs was expected in goats than in cattle. Indeed, the results presented here suggest that goats residing close to wildlife populations are not at a greater risk of FMD infection than those living further away.

### Transmission within the domestic compartment

Overall, the individual-level prevalence was lower in goats than in cattle. In terms of prevalence at different levels, the between-herd prevalence was lower in goats than in cattle, whereas the within-herd prevalence in positive herds was very similar in cattle and goats. This suggests that herd infections are less frequent in goats than in cattle, whereas once a herd is infected, the degree of infection spread within a herd does not differ between goats and cattle.

The difference in overall seroprevalence observed between the two species can be explained by three main hypotheses, which are presented here. However, validating any of these hypotheses would require further investigations. The first and most plausible hypothesis is that cattle, which have a larger home range than goats do, experience higher contact rates between herds, leading to a greater likelihood of exposure to the virus in cattle than in goats. The second hypothesis considers intrinsic host-specific factors to explain the seroprevalence differences. Variations in susceptibility, infectivity, or virus shedding capacity may contribute to the overall ability of each host species to participate in viral circulation and thereby be exposed during intraspecific contact [52]. However, a similar intra-herd prevalence was observed in both species, which does not support this second hypothesis. Indeed, if goats are less competent hosts than cattle are, a lower intra-herd prevalence would be expected. Finally, a third hypothesis suggests different immune responses between cattle and goats. If cattle produce antibodies over a longer period, their higher seroprevalence may reflect this. For example, the carrier stage lasts up to three years in cattle [53], whereas it lasts only four months in goats [54].

Further analyses revealed that there was a strong intraindividual correlation of serological status during the longitudinal survey. This finding indicates that individuals tend to maintain their serological status over time, whether positive or negative. At the intra-herd level, our results also revealed no evidence of a correlation for seropositivity in either cattle or goats within the studied areas. This suggests an absence of clustering of positive animals within herds for both species.

These findings imply that the relative contribution of intra-herd transmission to infection events might be less prominent than initially thought, particularly in goats. Moreover, our results confirm that seroprevalence in cattle and goats from the same farm—despite potentially having direct contact and sharing similar exposure risks—remains unrelated. This finding supports the hypothesis that interspecies transmission between cattle and goats is infrequent and that the infectious dynamics of the two species appear to be independent.

### Individual factors affect FMDV serodynamics in livestock

The intra- and interspecific differences in livestock seroprevalence can be explained by different species-specific animal management practices as well as physiological individual-based factors. The results from our study strongly support previous FMDV serological studies in cattle [7, 16] and the hypothesis that goats are suitable hosts for FMD virus and, consequently, have a function in multi-host system dynamics. The evidence of FMD virus circulation in goats in Zimbabwe is recent and has only been addressed in a serological investigation from a single region [23]. Across sites, male livestock individuals presented lower odds of being seropositive than females did. Males are generally separated from females and young animals in enclosures, reducing the likelihood of close, infectious contacts. In contrast, females interact with one another and with calves that, after the first six months of life and the waning of maternal antibodies, are immunologically naïve, thereby posing a risk of contributing actively to viral circulation. Age class was also a determining factor in explaining the serostatus of individuals. Animals under 6 months of age were not sampled, so the juvenile category refers to individuals who are theoretically no longer protected by maternal immunity and thus naïve to FMDV. During their first year, cattle calves are usually kept in enclosures separate from adults (except for their mothers) and have limited opportunities to mix with the herd or to be exposed to potential sources of infection (e.g., during dipping/vaccination sessions or at markets). As a result, adults may have higher seropositivity because they have had more frequent opportunities for exposure in recent months. This aligns with previous studies [53, 54]. However, this pattern was not detected in goats, which contrasts with studies indicating that older goats have a greater probability of being seropositive [55]. Unlike cattle, goats are generally managed in a more consistent manner throughout their lives, with unrestricted movement. Therefore, the probability of exposure to FMDV is likely more uniform over the lifespan. Our study underscores the differing risk of exposure among juveniles, subadults, and adults. However, the classification into broad age categories may have limited our ability to fully capture individual exposure histories. For example, goats, which are often slaughtered shortly after reaching adulthood, may not have experienced significantly different exposure opportunities than younger individuals did.

### Contrasting FMDV circulation patterns: site-specific insights

Our results also highlight significantly different FMDV circulation patterns between the two study sites (i.e., Hwange and Chiredzi SES), highlighting interesting elements for understanding the epidemiological dynamics in these systems. Different types of interfaces with PAs have already been shown to trigger higher FMDV prevalence in adjacent livestock populations as well as more outbreaks, as indicated by comparisons of the Chiredzi and Hwange SESs [7, 16]. Indeed, despite the similar climatic and ecological characteristics of semiarid savannah, the different distributions of resources (i.e., water and forage), which directly influence the movement of and contact patterns between reservoir and sensitive populations (i.e., cattle and buffalo [10]), are likely the cause of the differences observed in FMDV seroprevalence [16].

### Limits

As previously discussed, the presence of antibodies in an individual indicates a past infection. In this study, we rely on the prevalence of these antibodies to describe spatial patterns of infection and highlight risk factors. However, it is important to note that seroprevalence is only a proxy for individual virus exposure, and this link is complex and influenced by other factors that cannot be addressed through serological monitoring alone. Thus, the main limitation of this study, which prevents further exploration of the epidemiology of FMD, lies in the lack of knowledge regarding the lifespan of the antibodies we detected. Few studies provide a clear answer for our species of interest, and it is necessary to understand this pattern for each age class, sex, and any other individual factor that may affect an animal's health. On the other hand, both cattle and goats have the capacity to become carriers of the virus [13] and thus produce antibodies over a long period of time, ranging from 3 years for cattle [53] to 4 months for goats [54]. While data on the duration of post-FMDV infection antibody persistence are lacking, hindering us from conclusively determining virus circulation patterns, the presence of antibodies in juvenile individuals allows us to assert that livestock populations have recently been in contact with the virus at both study sites. Molecular and phylogenetic approaches for virus strains circulating in the populations of interest could provide key elements to support or contradict our transmission pathway hypothesis. Such results would also enable us to address crucial questions within this framework, such as host susceptibility and their ability to excrete the virus into the environment.

### General conclusion

Here, we present the first longitudinal serological monitoring of FMD conducted on goats and cattle simultaneously in southern Africa. Not only does this monitoring provide robust evidence to confirm the ability of goats to circulate FMDV within the community, but it also presents evidence that the infection dynamics observed in goats and cattle follow different spatial patterns. These findings could serve as a solid foundation for the assertion that the two livestock species belong to distinct epidemiological populations with specific functions in the transmission chain. In the literature, cattle are considered efficient hosts for FMDV circulation [55]. In the specific context of our SES in Zimbabwe, we observed serological patterns that suggest that infections by cattle may not be the sole or even the main driver of FMDV circulation in the domestic compartment. However, the higher between-herd prevalence in cattle than in goats suggests that the cattle population plays a more important role in maintaining the virus in the livestock compartment than does goats. Our findings do not seem to substantiate any discernible correlation between the virus's circulation among goats and their interaction with either wild host populations or cattle sharing the same habitat. The virus may circulate within goat populations, primarily through intra-herd transmission and infrequent inter-herd transmission. Characterizing the viral strains circulating in the different populations and species would allow these hypotheses to be tested on the role of the different species in the community.

Finally, this work emphasizes the importance of conducting comprehensive surveillance routines for multiple sympatric species in the case of multi-host pathogens [56]. This study adds to the body of literature supporting the premise that high-risk contacts should be central to environmentally sustainable disease control within these interface zones, involving local stakeholders from agricultural and protected areas. It is also imperative to address the epidemiological dynamics within livestock populations, which may contribute to the inefficacy of disease management actions, largely focused on restricting interactions between wild and domestic hosts. Until a detailed and context-specific description of the role of each domestic and wild animal population in each FMD system is provided, claiming a thorough understanding of infectious dynamics at the wild–domestic interface remains ambitious.

## Data Availability

The datasets analysed during the current study are available from the corresponding authors upon request.
